# General Strategies for Preparing Hybrid Polymer/Quantum Dot Nanocomposites for Color Conversion

**DOI:** 10.3390/nano13233072

**Published:** 2023-12-03

**Authors:** Guan-Hong Chen, Chen-Te Lin, Po-Hsun Chen, Tyng-Woei Jang, Hsueh-Shih Chen

**Affiliations:** 1 Department of Materials Science and Engineering, National Tsing Hua University, Hsinchu 30013, Taiwan; p01122334@gmail.com; 2 College of Engineering, National Tsing Hua University, Hsinchu 30013, Taiwan; chentelin@gapp.nthu.edu.tw (C.-T.L.); xenic0413@gmail.com (P.-H.C.); mse111031624@gapp.nthu.edu.tw (T.-W.J.); 3 College of Semiconductor Research, National Tsing Hua University, Hsinchu 30013, Taiwan

**Keywords:** quantum dots, acrylate, silica–titania, sol–gel, light extraction, display

## Abstract

Quantum dots (QDs), with their exceptional optical properties, have emerged as promising candidates to replace traditional phosphors in lighting and display technologies. This study delves into the integration strategies of QDs within glass and polymer matrices to engineer advanced quantum dot color converters (QDCCs) at the industrial scale for practical applications. To achieve enhancements in the photostability and thermal stability of QDCCs, we explore two distinct approaches: the dispersion of QDs in a hydrophilic glass matrix via a sol–gel process and the incorporation of QDs into a non-polar acrylate monomer to formulate QD/polymer nanocomposites. This research further investigates the optical behaviors of these composites, focusing on their light-scattering and propagation mechanisms, which are critical for optimizing light extraction efficiency in QDCCs. Additional optical film and light-scattering particles can improve color conversion efficiency by ~140%. These advancements present a significant step forward in the development of high-performance, energy-efficient, QD-based lighting and display systems.

## 1. Introduction

Solid-state light-emitting diodes (LEDs) are extensively used in both human lighting solutions and the backlighting of consumer electronic products, owing to their cost-effectiveness, energy efficiency, and high reliability [1,2,3,4]. Compared to conventional luminescent materials such as inorganic phosphors and organic dyes [5,6,7], quantum dots (QDs) have emerged as promising down-convertors because of their size-tunable colors, narrow emission bandwidth, and wide absorption spectrum [8,9,10,11,12]. However, QDs are reactive to moisture, heat, and radiant fluxes, so transparent materials have been employed to encapsulate QDs, which are referred to as QD color converters (QDCCs) and have been launched in various applications [13,14,15,16,17,18].

Previous studies were focused on advancing film coating and pixilation methods, aiming to bring QDCCs into the realm of microdisplays. Several perspectives are being considered for developing QDCCs, including the (1) dispersion of QDs in a transparent medium; (2) the optical performance of QDCCs; (3) the quantification of QD solid content; and (4) the design of light extraction. In terms of QD dispersion, the surface chemistry of QDs is rather complex, and QDs tend to aggregate in a matrix due to chemical compatibility and/or phase separation [19,20]. The shortened inter-distance between QDs may seriously cause optical reabsorption and induce the nonradiative fluorescence resonance energy transfer (FRET) process; this can significantly decrease the conversion efficiency of QDCCs [21,22], which is considered a key factor in examining the performance of QDCC products. Regarding the optical performance of QD films utilized in display backlighting, traditional optical measurement methods often lead to ambiguity when dealing with high concentration QDCCs, e.g., >30 wt.%. Moreover, quantifying the solid content of QDs using traditional gravimetric methods proves challenging and unreliable. Variations in concentration due to existing ligands can notably affect the optical properties of QDCCs. For the design of light extraction, light guiding and/or light recycling may be required, as the optical absorption cross-sectional area of nano-sized QDs is generally limited, e.g., <20 nm.

In this study, we comprehensively discuss the experimental strategies for preparing an applicable QDCC. Among the experimental steps, the chemical compatibility between QDs and the selected matrix is thought to be one of the most important factors. Using a proper monomer to introduce QDs in a hydrophobic polymer precursor solution affects their dispersion, and thus, optical performance in QDCC products. Moreover, various factors and concerns in preparing high-performance QDCCs are discussed and suggested. For example, the optical performance of QDCCs is alternatively evaluated by using so-called light conversion efficiency (LCE) instead of absolute quantum yield (QY_a_), which aims to minimize experimental errors when comparing luminescence efficiency in different batches. Optical absorbance is used to represent the QD concentrations instead of weight measurements, reducing the instrumental errors in different batches. While the optical absorption of QDCCs can be enhanced by simply introducing scattering particles, additional proper optical prism films may be simultaneously utilized to improve light extraction. All the polymers investigated are available at the industrial scale in this study. 

## 2. Materials and Methods

### 2.1. Materials

Green (G) and red (R) ZnCdSeS alloyed QDs were provided by Tsing-Yang Technology Inc. Tetraethoxysilane (TEOS, 99%), titanium isopropoxide (TTIP, 97%), anhydrous methanol, absolute ethanol, 1-propanol, tetramethylammonium hydroxide (TMAOH) in methanol, NH_4_OH, and (3-mercaptopropyl)trimethoxysilane (3-MPS) were purchased from Sigma-Aldrich (St. Louis, MO, USA). All reagents were used without any further purification. Deionized water was used to initiate hydrolysis and condensation reactions. Polystyrene (PS) microparticles with a diameter of 5 μm were purchased from Soken Chemical & Engineering™ Co., Ltd, Tokyo, Japan. Phenylbis(2,4,6-trimethylbenzoyl) phosphineoxide (BAPO) and trimethylopropane trimethacrylate (TMPTMA) were purchased from Eternal Materials Co., Ltd, Taiwan.

### 2.2. Surface Modification of QDs and Assembly of QD/MPS in SiTiO_x_ Beads

The modification of oleic acid-capped QDs by 3-MPS was performed using the following method: 10 mg of QDs in toluene was precipitated by adding a non-solvent (methanol) to remove excess ligand. The wet precipitate was dispersed in 50 μL of 3-MPS. After shaking and ultrasonication for 5 min, 500 μL of TMAOH methanol solution (pH 10) was added to deprotonate the thiol groups. The optically clear mixture was diluted with 10 mL of methanol. After 1 h of stirring under an inert atmosphere, the MPS-modified QDs (QD/MPS) were precipitated by toluene. The precipitate was dispersed in methanol.

The preparation of a SiTiO_x_ (STO) precursor sol solution was modified from a previous work, which was prepared by mixing pre-hydrolyzed silica and titania precursor solutions [23,24]. The partly hydrolyzed TiO_x_ and SiO_x_ sols were mixed to form a SiO_x_–TiO_x_ precursor sol solution that was colloidally stable for months. The formation of QD/MPS/STO particles was modified via the typical synthesis of sol–gel nanoparticles [13,14]. The as-prepared QD/MPS in methanol was precipitated by toluene. The wet precipitate was dispersed in the as-prepared STO sol. An optically clear colloidal solution of QD/MPS-STO mixture was easily obtained after ultrasonication for 60 s. Then, the mixture was diluted tenfold by adding additional methanol, and basified to ~pH 10 with ammonia. To grow the STO shell on QD/MPS, doubly distilled water was slowly added to the mixture at room temperature. Typically, the molar ratios of QDs, STO, methanol, and H_2_O were 1, 2 × 10^5^, 7 × 10^7^, and 2 × 10^7^. After 3 h, QDs@STO beads were separated from the solution via centrifugation. The beads were re-dispersed in ethanol for further characterization.

### 2.3. Preparation of QD/Polymer Color-Enhanced Films

The preparation of QD/acrylate color-enhanced films was modified from previous work [25,26]. The precursor of QD films was prepared by re-dispersing commercial green and red QDs (λ_em_ at 530 nm and 620 nm) in a UV-curable acrylic resin (solid content of QDs ~1 wt.%). The resin formula comprised a 45 wt.% acrylic monomer, 15 wt.% TMPTMA as a crosslinker, 2 wt.% BAPOs as a photoinitiator, 3 wt.% PS scattering particles, and 35 wt.% urethane acrylate oligomers to increase viscosity. The mixing process was performed using a dispersing instrument (T10 basic ULTRA-TURRAX^®^ from IKA-Werke GmbH & CO. KG, Stauffen, Germany). The QD-acrylate mixture was then coated on a PET substrate (thickness 100 μm) using a blade coater, followed by laminating with a cover PET and curing under a 500 W high-pressure mercury lamp. The total thickness of QD/polymer films with two PET sheets was approximately ~320 μm (10 × 10 cm^2^).

### 2.4. Characterization

The photoluminescence (PL) properties of QDCCs were recorded using a fluorescence spectrometer, and the QY_a_ of QD solutions were measured within an integrating sphere (Horiba FluoroMax-4, Kyoto, Japan). The light emission spectra, chromaticity, and LCE of QD films and QD-converted LED were measured via fluorescence spectrometry (RLS-1000, Rainbow Light Optical Co., Ltd, Taoyuan, Taiwan), and the QD film samples were assembled in a modified 15.0” LCD panel with blue excitation (15.0” AUO G150XG03) in the structure of metal reflector/light guide plate (LGP)/QD film/brightness enhancement films (BEFs). The chemical composition of samples was analyzed via thermogravimetric analysis (TGA, Q500, TA Instruments, New Castle, DE, USA) and inductively coupled plasma mass spectrometry (ICP-MS, iCAP TQ ICP-MS, Thermo Fisher Scientific, Karlsruhe, Germany). The optical density of QD solutions was measured using a UV-Vis/NIR spectrophotometer (JASCO V-700, Osaka, Japan).

## 3. Results and Discussion

### 3.1. QDs in a Transparent Matrix

#### 3.1.1. QDs in Hydrophobic/Hydrophilic Polymer Matrix

In the development of QDCC, QDs and a transparent matrix are essential components. The transparent matrix serves two primary purposes: (1) offering sufficient distance for QDs to prevent strong re-absorption from aggregation, and (2) providing a gas barrier to protect QDs against environmental influence. These transparent matrices can be composed of either organic or inorganic materials, such as polymers, epoxies, and silicon resins, which are widely utilized in the industry. Among these options, acrylic-based resin has been a prevailing choice in the industry for several decades due to features such as its low cost, transparency, and versatility in adjustable functionality. Notably, acrylic resin exhibits excellent chemical compatibility with the hydrophobic nature of QDs, facilitating their effective incorporation with QDs in various applications [27,28].

The formulation of acrylate-based QDCCs involves various processes, such as formulation design, mixing, coating, curing, and film formation. Acrylic resin typically comprises a monomer solution for viscosity adjustment, an oligomer for mechanical properties, a crosslinker for environmental resistance, and a photoinitiator (PI) for the photo-curing procedure. The nuanced balance among these components significantly influences the characteristics of the synthetic photopolymer and, most importantly, directly impacts the optical performance of the embedded QDs. Considering the high viscosity (>3000 cp) of oligomers in the formula, which can make it challenging to homogeneously mix QDs in a viscous resin through physical approaches (e.g., vortex or rotation stirring), an alternative approach is employed. QDs are initially dispersed in the liquid monomers, allowing the monomers to effectively bring the QDs into the acrylic resin. Therefore, this highlights that the mixing process is one of the most critical steps in the fabrication of acrylate-based QDCCs. Several factors must be carefully considered, including the chemical compatibility between materials, the colloid properties of the QDs, and the polymerization of the resin, in both the monomer solution and the mixing resin solution.

**Figure 1:**
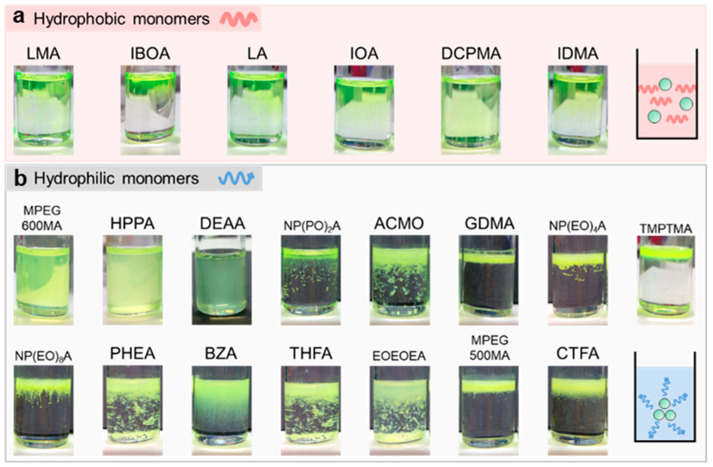
Photos of QDs dispersed in hydrophobic (**a**) and hydrophilic (**b**) acrylic monomers. Right inset schemes illustrate the QD particles dispersed in the monomers. Full names of the monomers are listed in Table S1.

**Figure 2:**
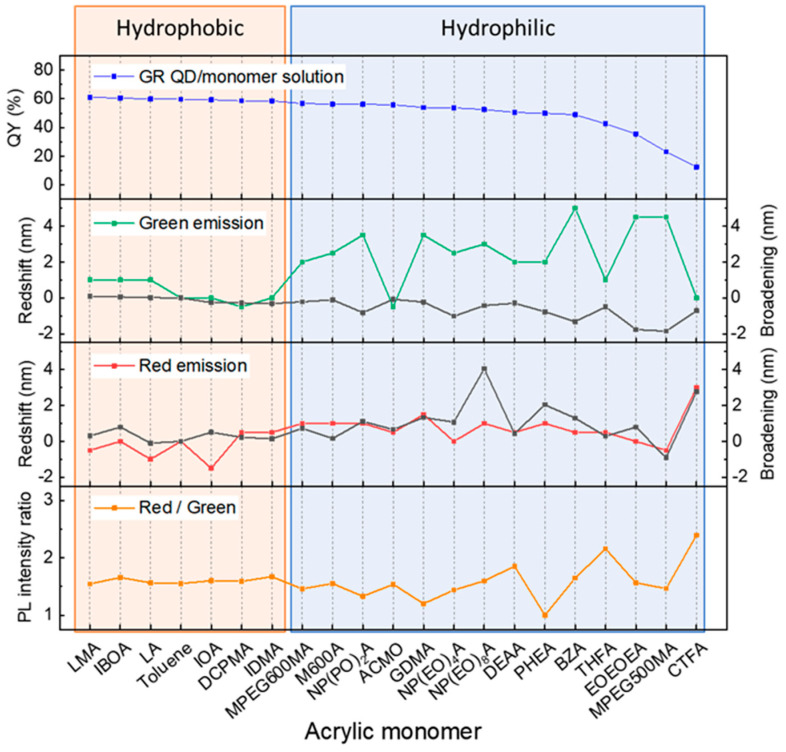
PL properties of QD/monomer mixtures, including QY, peak shift, peak broadening, and the integration intensity ratio of the PL bands. The mixtures contain green and red QDs.

Figure 1 shows the dispersion of QDs within various monomers obtained from industrial suppliers, each with different functional groups (as summarized in Table S1). The hydrophobic monomer features hydrophobic functional groups, such as long alkyl chains, and has minimal solubility in water (<0.2 mg/L). In contrast, the hydrophilic monomer possesses hydrophilic functional groups (e.g., a hydroxyl group) and is soluble in water. Since QDs are typically synthesized in an oil-based environment, a hydrophobic nature is conferred by surface-passivated ligands with long alkyl chains, such as oleate, phosphine, or thiol. It is noted that the dispersion mixtures visually exhibit better colloidal properties when QDs are dispersed in hydrophobic monomers. Among these hydrophobic monomers, lauryl methacrylate (LMA) and isobornyl acrylate (IBOA), distinguished by their good chemical compatibility with QDs, successfully achieve homogeneous QD dispersion. These observations clearly establish a correlation between dispersion quality and optical performance. The optical properties of these QD/monomer mixtures are plotted in Figure 2 (details are summarized in Table S2). The QDs dispersed in the hydrophobic monomers show a high QY without the PL peak shifting or broadening, indicating that hydrophobic monomers serve as a suitable QD medium similar to a non-polar organic solvent (e.g., toluene). In contrast, dispersing QDs in hydrophilic monomers results in a lower QY, accompanied by the PL peak shifting and broadening, along with visible aggregation or agglomeration. The QY declines as the increased R/G intensity ratio implies reabsorption between red and green QDs. On the other hand, it suggests a reduction in the inter-distance between QDs, typically associated with FRET behavior induced by QD aggregation.

**Figure 3:**
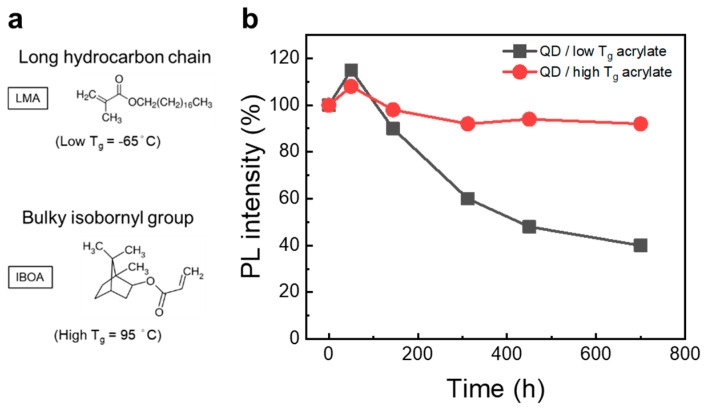
Effect of polymer structures on the thermal stability of QDCCs. (**a**) Molecular structures of two representative acrylic monomers: low-T_g_ LMA and high-T_g_ IBOA. (**b**) Ex situ PL intensity trace of thermal stability for QDCCs directly heated at 90 °C in atmosphere.

Based on the meticulous selection and testing of various monomers, as described above, we demonstrated that acrylate-based QDCCs show good optical performance (Figure S1). These monomers with different hydrophobic molecular structures show a great influence on the thermal stability of QD/polymer films. For instance, LMA and IBOA monomers are commonly used in industry, each possessing a distinct glass transition temperature (T_g_) attributed to their respective polymer structures, as shown in Figure 3a. The T_g_ of LMA and IBOA is characterized to be −50 and 80 °C, respectively. The thermal stabilities of the acrylate-based QDCCs using LMA and IBOA as monomers (45 wt.%) in the matrix are shown in Figure 3b. Notably, the QD/IBOA film retains 90% PL intensity even after being placed in a 90 °C oven for 700 h, indicating that the high-T_g_ matrix provided better thermal stability. The high value of T_g_ is correlated with the mobility of the polymer chain, where the bulky and rigid bicyclic isobornyl group of IBOA may hinder the movement of molecules in the polymer as well as the permeation of oxygen and water molecules.

#### 3.1.2. Chemical Modification of QDs in Hydrophilic Silica–Titania Glass

It is widely *agreed upon* that the surface chemistry of QDs is highly sensitive to environment species such as oxygen and moisture. QDs without proper protection are susceptible to oxidation, leading to structural degradation and decreased fluorescence intensity [29]. Our recent findings suggest that even a polymer matrix may offer only temporary protection against the oxidation process [30,31]. Rather than dealing with the complex photochemical reactions occurring at the QD surface, an alternative approach involves overcoating with an inorganic layer to inhibit QD oxidation [32,33]. Furthermore, the use of an inorganic silica overcoating, which is a transparent matrix exhibiting a hydrophilic nature, is highly compatible with silicone matrices [34,35]. In general, QDCCs directly incorporated into an LED package show a relatively short lifetime [36,37], likely due to the dissociation of organic ligands from the QD surface caused by photo and/or thermal effects during device operation [38,39]. Thiol-functionalized or silica-shelled QDs are known for their enhanced stability compared to phosphine-passivated QDs [40]. 

**Figure 4:**
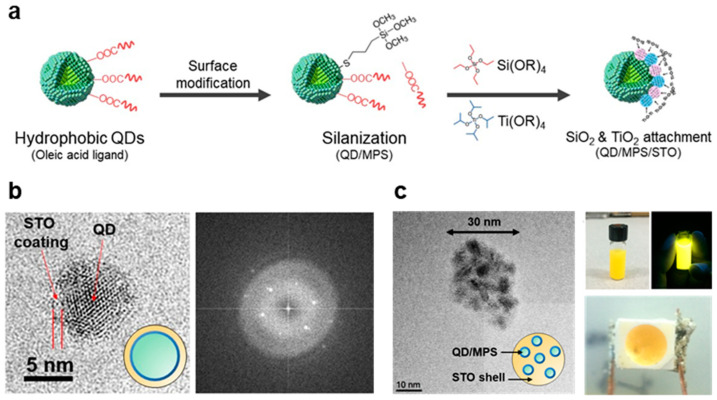
(**a**) Surface modification of QDs with 3-MPS as a silane coupling agent and the attachment of a silica and titania shell through hydrophilic sol–gel process. (**b**) TEM image of a QD encapsulated with an STO glass shell, where the coating thickness is 1–2 nm. TEM diffraction pattern of the crystalline QDs and STO coating. (**c**) TEM images of QDs@STO beads derived from the QD/MPS/STO sol–gel solution. The top-right photos show the QDs@STO beads dispersed in ethanol under room light and UV. The bottom-right photo is a surface-mount device (SMT) QD LED with QDs@STO beads as the QDCC in silicone resin on a blue chip (on-chip structure).

Typically, silica obtained through the conventional sol–gel process features micropores, and its microstructure is rather loosely organized. Prior research has indicated that silica–titania (STO) glass presents a more compact microstructure, where titania molecules can occupy the micropores within the silica and stabilize the Si-O-Si bonds [23]. Sol–gel STO glass has been utilized to encapsulate inorganic nanoparticles [24]. To incorporate hydrophobic QDs in the sol–gel STO process, the QD surface should be chemically modified, as shown in Figure 4a. Silanes can be utilized as hydrophilic ligands to replace the hydrophobic ligands of QDs. In this study, silanes with thiol groups (e.g., 3-MPS) are considered as promising coupling agents because of the inherently strong interaction between thiol compounds (–SH or mercaptans) and QD surfaces [41]. The thiol groups can readily replace the original ligands on the QD surface at room temperature without causing significant deterioration or oxidation [42,43]. The hydrolysis of silane coupling agents allows the modified QDs (referred to as QD/MPS) to be well dispersed in a polar solvent like ethanol (Figure S2). The resulting QD solution exhibits a good colloidal solution with a clear and transparent appearance, indicating that the successful modification of the QD surface through the hydrolysis of the 3-MPS ligands has led it to develop a hydrophilic nature. Moreover, MPS ligands on the QD surface could be beneficial for the growth of shells composed of Si and Ti alkoxides (e.g., TTIP and TEOS), as illustrated in Figure 4b. The formation of an STO overcoat is confirmed by TEM images and diffraction patterns. This overcoat enhanced the environmental resistance of QDs and enabled adjustments to the refractive index. 

**Figure 5:**
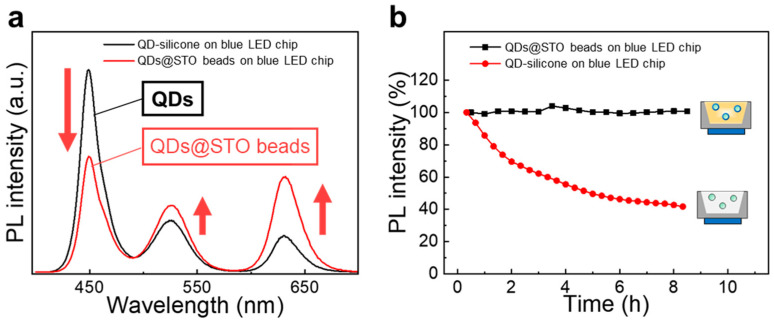
(**a**) PL spectra of pristine QDs and QDs@STO beads excited by blue light LEDs. The QDCC with QDs@STO beads shows a 115% increase in illuminance. The QD concentration is fixed at 1 wt.% in the LED package. (**b**) PL intensity stability of pristine QDs and QDs@STO beads excited by blue light LEDs. The PL intensities are normalized to the initial value (0 h).

Multiple QDs can be encapsulated in a nanosized STO bead (denoted as QDs@STO) from the QD/MPS/STO solution by varying the QD particle concentration, as shown in Figure 4c. The QDs@STO beads, considered to be light diffusers, are found to enhance the light scattering and blue light absorption in optical structures such as film or LED packages, as shown in Figure 5a. Compared with pristine QDs, QDCCs involving QDs@STO beads can more efficiently convert the blue chip light in a QD LED, resulting in easily obtaining white light in the 1931 CIE color diagram (Figure S3b). Moreover, QDs encapsulated in STO shows improved photostability, as shown in Figure 5b. The QDs@STO-based QDCCs demonstrate a limited decrease in PL intensity after an 8 h photoaging process, indicating that the MPS-modified QDs encapsulated in the sol–gel STO glass exhibit high stability against the photoaging process. This result suggests that QDs encapsulated in a dense matrix such as STO glass may be a solution to the current challenges in QDCC applications.

### 3.2. Preparation of QDCC for Lighting and Displays

#### 3.2.1. Calculations of Luminescence Efficiency of QDCCs

**Figure 6:**
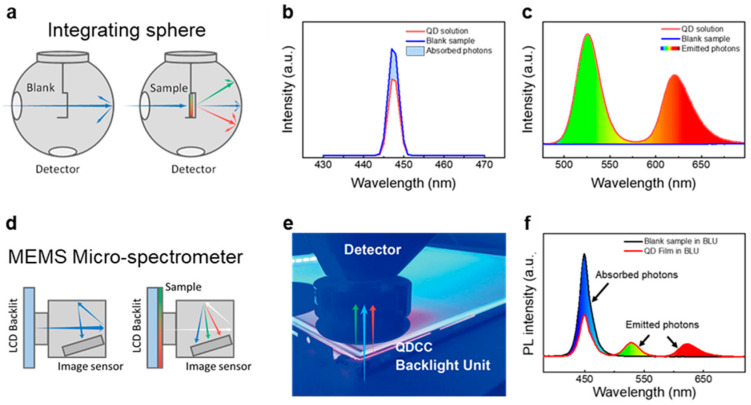
(**a**) Schematic diagram of QY_a_ measurement. (**b**) Absorption of exiting photon (blue area), and (**c**) integrating area of green and red QD emissions. (**d**) Schematic diagram of LCE measurement. Compared to an integrating sphere, this approach allows for easy switching and an appropriate opening size for both the backlights and QDCCs. The MEMS micro-spectrometer enables rapid collection of the PL spectra and the direct outputting of various derived values. (**e**) Photo of LCE measurement of QDCC stacked between the optical films of a 15” backlit LCD. (**f**) The collected PL spectrs of backlit LCD (reference) and QDCCs (sample). LCE is calculated as the ratio of QD emission area to reduced excitation area (blue light, in this case).

QY_a_ measurement using an integrating sphere is a proper way to obtain luminescence efficiency, because the relative QY (QY_r_) method needs to compare a fluorescent standard with a known QY that is usually an organic dye (Figure 6a–c). However, inaccuracies or mistakes usually occur in the QY_r_ when an organic dye rapidly degrades, so its PL QY significantly decreases. In instances where QY_r_ is measured over time, the observed QY_r_ can be expectedly high if directly compared with a decayed standard, especially when the dye stock solution has been stored for an extended period, like several days, in an atmospheric environment. Inconsistent PL QY in QD solutions is often identified as overexposure of the background in photographic images of QD samples in some reports. For a QY_a_ measurement, it is defined as the ratio of the photons emitted by the luminescent materials over the absorbed photons. To obtain a reliable PL QY_a_ measurement for QDs, the QD concentration is an important factor to consider. The optical density of the QD solution is usually kept at least below 0.02 to mitigate the effects of reabsorption, which can occur at high QD concentrations and may lead to underestimation of the QY_a_. In contrast, QDCCs are manufactured in versatile forms, such as powders, inks, and films, usually containing concentrated QDs where high optical density can easily exceed the threshold for accurate PL QY_a_ measurement, leading to distortions in efficiency. It is important to note that only the forward optical pathlength orthogonal to the QD film is efficient for displays; the efficiency collected from the integrated sphere may be overestimated. Therefore, there is an urgent need for a rapid and reliable measurement method that offers flexibility in adapting to various forms of QDCCs and backlights, especially considering the fast-changing nature of the display industry.

In the proposed measurement set up, an LCD panel backlit with blue light emission is assigned as a reference. When QDCC is placed onto the backlit panel, the QD-converted light and the unabsorbed transmitted backlight are collected by a microelectromechanical system (MEMS) micro-spectrometer, as shown in Figure 6d. Through data collection with a MEMS micro-spectrometer, a convenient and practical spectrum measurement method is established, as shown in Figure 6e. The estimation of LCE is defined as the ratio of the QD emission peak area to the reduced blue peak area (Area_reference @450 nm_–Area_QD film @450 nm_), as illustrated in Figure 6f [29]. The output values can be programmed to directly demonstrate the calculated efficiency, luminous efficacy, coordinate point, and other derived parameters. This method is not constrained by the size or optical design of QDCC-based products. Moreover, an additional advantage is the cost-effectiveness of employing a MEMS micro-spectrometer compared to an integrating sphere.

#### 3.2.2. Quantification of QD via Gravimetric and Optical Methods

**Figure 7:**
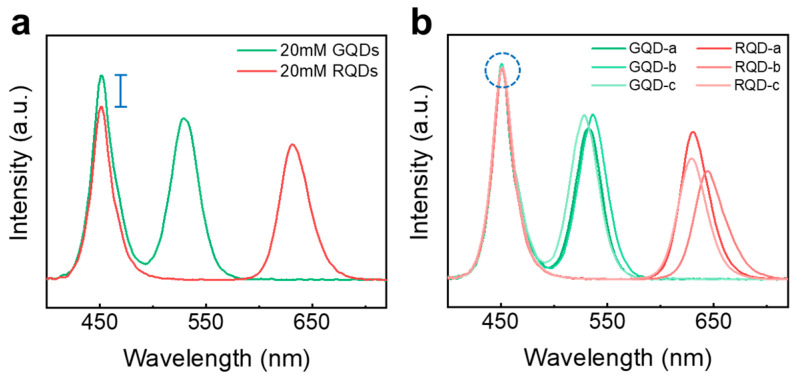
Absorption controls (at 450 nm) of QD films with different QDs when using the same (**a**) molarity (Gravimetric quantification of QD) and (**b**) optical density (Linear absorption evolution) of QD for concentration quantifications.

In the evaluation of QDCCs, two key optical performances are LCE and optical density, which, respectively, govern fluorescence intensity and color purity. Since QDCCs are required to efficiently convert high-energy monochromatic backlight (violet or blue) into low-energy monochromatic light (blue or green/red), the proportion of transmitted backlight significantly impacts color purity. The concentration of QDs, as the most crucial variable in the formulation, determines the number of absorbed photons within an optical medium. In the conventional pigment industry, ingredients are directly specified and quantified by their weight or volume (i.e., wt.% or vol.%) as gravimetric concentrations in the formulation. However, nanosized QDs pose a challenge when attempting to accurately measure their tare weight without interference from moisture or residues, especially when employing advanced precision balances. In terms of currently existing QD color-enhanced films, only a small quantity of QDs are required within the optical medium to effectively absorb and convert blue light sources, aiming for enhanced environmental sustainability. For instance, it consumes 4–8 mg of QDs in a 6 × 6 cm of QD color-enhanced film with a concentration of 0.4–0.8 wt.%. This value is almost within the error margin for the weighing instrument (with a weighing reliability of 1–0.1 mg). Even slight variations in the weight of QDs within final products can result in significant deviations in backlight consumption, as shown in Figure 7a.

In contrast, a dilute QD solution exhibits linear absorption behavior at specific wavelengths as the QD concentration increases (Figure S4). With the absolute concentration carefully estimated via the conventional gravimetric method (Figure S5), it is possible to determine the absorption coefficient of QDs, represented by the slope of the curve when using Beer’s Law. Consequently, an alternative quantification method based on the optical absorption of light-absorbing materials is developed. For QDs of similar emission wavelengths, we can estimate their solution concentration by measuring their optical density at different dilution levels. The quantified QDs used as reference samples can be further employed to estimate the relative concentration of different QDs, for which the concentration of QDs is unknown, showing a linear relationship between absorption and different concentration levels. The difference between the intercepts (ΔA_reference_ and ΔA_unknown_) represents the variance in the number of photons absorbed at specific wavelengths, facilitating the evaluation of the relative concentration of unknown QD solutions with regard to the reference QDs (Figure S4b). The quantification method based on photon absorption ensures excellent reproducibility of blue absorption among QDCCs utilizing different QDs, as shown in Figure 7b. By simplifying concerns regarding complex effects depending on wavelength and concentration, this method proves beneficial for tailoring the PL spectrum and fine-tuning the color space in display applications, resulting in highly reproducible and adjustable color characteristics of QDCCs.

#### 3.2.3. Effect of QD Concentration and Film Thickness in QDCCs’ Optical Performance

**Figure 8:**
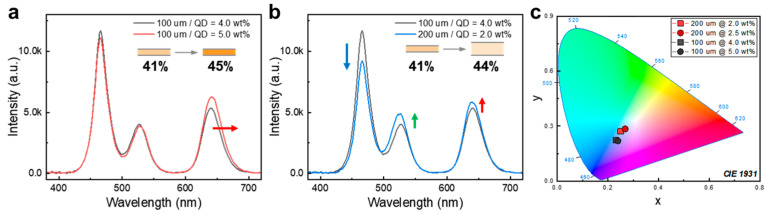
Effects of (**a**) QD concentration and (**b**) QD film thickness on (**c**) color coordinates in CIE color space. Inset schemes show the corresponding film efficiencies.

In conventional phosphor lighting applications, it has been observed that high-concentration phosphors can intensify the output emission flux and shift to desired color coordinates [44,45,46]. However, when using QDs as novel phosphors, a high concentration leads to the opposite result with a significant redshift of PL and loss of emission efficiency. This event is attributed to Forster energy transfer between aggregated QDs, as well as the overlap of absorption and emission spectra, leading to self-absorption and suppressed light output within the optical medium of QDCCs [47]. Figure 8a shows the change in PL spectra as the QD concentration increases from 4 to 5 wt.% in the QD films for a wide-gamut backlit LCD. While the LCE of QDCCs increases from 41% to 45%, the slight improvement in blue absorption but redshift in red emission imply the occurrence of reabsorption due to the reduced distance between QDs. When the inter-distance of QDs is expanded by both reducing the QD concentration (from 4 wt.% to 2 wt.%) and increasing the film thickness (from 100 μm to 200 μm), blue absorption is markedly improved without the undesired reabsorption, as shown in Figure 8b. These results are attributed to the extended optical pathlength for blue light within the medium, effectively mitigating light trapping and increasing the probability of QD light absorption. The improvement in blue light absorption then leads to more efficient QD emission, making it easier to reach the white point in a red/green QDCC-based white LED with a lower QD concentration, as shown in Figure 8c.

It may be concluded that the PL properties of QDCCs are conjointly determined by various factors, including the thickness of the optical medium and the features of the QDs (e.g., concentration, QY, and size). Considering industrial samples used in LCD displays, QD color-enhanced films generally consist of 0.5–2 wt.% QDs in film thickness ranges from 50 to 100 μm [25,48]. For the QDCCs applied in micro-displays, our recent developments have involved increasing the QD concentration to 30–40 wt.% along with significantly reducing the film thickness to less than 10 μm [49,50]. Interestingly, it has been observed that QD concentration is correlated with photostability to some extent. When a thin film has a high concentration of QDs, a greater number of QDs are positioned at the forefront, directly exposed to the blue light source. Those QDs situated deeper within the film, on the side opposite to the exposure, are likely to be shielded by the QDs at the front. Consequently, these shielded QDs can serve as a secondary wave of active material once the QDs at the forefront have degraded. Research on QD-concentration-dependent photostability should be carefully considered and is being conducted.

#### 3.2.4. Light Extraction of QD/Polymer Nanocomposites

**Figure 9:**
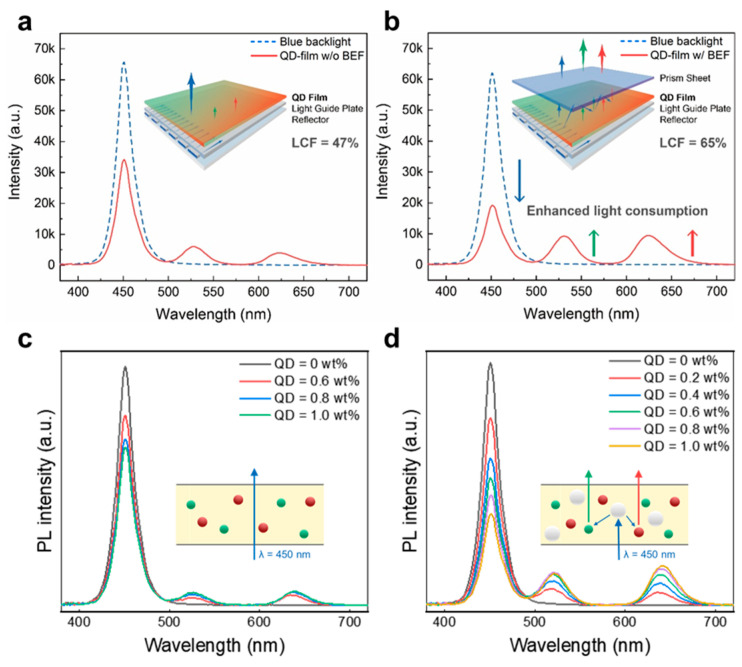
Strategies for light extraction in QDCCs. PL spectra of QDCC films without (**a**) and with (**b**) BEFs in the backlit LCD. PL spectra of QDCC films without (**c**) and with (**d**) light-scattering particles. QD concentrations in these films range from 0 to 1 wt.%. The inset schematic illustration depicts the respective effects of increasing the optical pathlength of incident excited light.

One of the challenges that QDCCs face is their poor blue light absorption because of lower absorbance in the blue range and the nanosized absorption cross-section of QDs. Careful design of the optical geometry and light scattering can enhance the extraction efficiency in QD-based lighting and display devices [51,52,53,54]. These techniques may involve incorporating additional BEFs or LSPs within the QD film to enhance their absorption capacity by increasing the optical pathlength. In conventional LCD displays, BEFs represent a key component for enhancing output brightness, managing the angular output of backlight by utilizing prismatic structures to focus light towards display viewers. When introducing BEFs into QDCCs, prismatic optics induced a light recycling phenomenon by extending the optic pathlength of incident blue light. Compared to backlit displays without BEFs (Figure 9a), Figure 9b shows that QDCCs equipped with BEFs exhibit significantly improved blue absorption without peak shifting or broadening in the PL spectra, resulting in a substantial increase in LCE from 47% to 65%. The effect of light recycling by additional optical films highlights the importance of developing QDCCs based on well-designed geometries of optical pathlength that may offer distinct improvements and potential benefits. Another optional technology for increasing the optical pathlength is light-scattering techniques, which are commonly employed to ensure the uniformity of light emission in optical applications [55,56]. Figure 9c shows the inefficient blue light absorption and minimal emission intensity when only QDs are dispersed in a transparent matrix. As the LSPs are added to QDCCs, the PL spectrum exhibits a significant increase in emission intensity and a noticeable enhancement in blue absorption, as shown in Figure 9d. This leads to a roughly 140% enhancement in LCE from 31.8% to 44.8%. The incorporation of LSPs into QDCCs shows an important effect on both backlight consumption and emission intensity enhancement. It is noted that the dispersion of LSPs also plays a vital role, similar to that of QDs dispersed in an optical medium. The presence of multiple particles system, with both R/G QDs and LSPs coexisting in the optical medium, raises complexities in the design, mixing, and operation of QDCCs. 

## 4. Conclusions

In summary, this research has formulated strategies for QDCCs in lighting and display applications. The fabrication process involves incorporating QDs into both glass and polymer matrices, suitable for different solution processes. This study reports that QDs exhibit chemical compatibility with hydrophobic monomers, and polymers with high glass transition temperatures act as efficient gas barriers for QDCCs, improving thermal stability, with T_90_ exceeding 700 h at 90 °C. Surface modifications of QDs enable their dispersion in hydrophilic solutions. QDs@STO beads exhibit a 115% enhancement in emission intensity, a 30−40% improvement in blue light absorption, and minimal photo-decay in on-chip QD-LED applications. Practical application is facilitated by using a micro-spectrometer for efficient and reliable measurements, and the proposed quantification method improves reproducibility and color calibration. Finally, for optimizing light extraction, the addition of optical films or light-scattering particles to QDCCs is recommended, resulting in an approximate 140% improvement in color conversion efficiency in both approaches.

## Data Availability

No new data were created or analyzed in this study. Data sharing is not applicable to this article.

## Supplementary Materials

The following supporting information can be downloaded at: https://www.mdpi.com/article/10.3390/nano13233072/s1. Figure S1. (a) PL spectrum of QD/acrylate optical film measured and integrated in a 15” blue LCD backlit. Inset photos of QD/acrylate optical film under indoor light (left) and UV lamp (right). (b) Thermal stability of QD solution and QD/acrylate film. Figure S2. (a) Photos of MPS-modified QDs highly-dispersed in ethanol solution. (b) QYs of as-synthesized QDs, QD/MPS and QD/MPS/STO. Figure S3. (a) Schematic diagram of QD optical diffusors for white LED compared with directly dispensing QDs in resin and showing longer light path length. (b) White points in CIE 1931 color space of QDCC-based on-chip LEDs along with the as-synthesis QDs (black point) and the QD optical diffusors (red point). Figure S4. (a) Linear absorption evolution upon the increasing QD concentration of QD toluene solution. (b) Estimation of absorption coefficient for optical density of QDs. Figure S5. Gravimetric quantification of QD solution using TGA and ICP-MS. (a) TGA measurement of the solid content of 50 uL QDs. The solvent is removed before TGA analysis. (b) Atomic ratio of QDs estimated from the ICP-MS. The QD molar mass is calculated according to the atomic ratio. (c) Calculation of QD molarity in solution by incorporating the data from TGA and ICP-MS. Table S1. List of acrylic monomers used in this study and their solubility in water. Table S2. Changes in PL properties of green and red QDs dispersed in different acrylic monomers.
