# Supplementary material for: General Strategies for Preparing Hybrid Polymer/Quantum Dot Nanocomposites for Color Conversion
Source: Nanomaterials (Basel). 2023 Dec 3;13(23):3072. doi: 10.3390/nano13233072 (PMC10708347; doi:10.3390/nano13233072)
Supplement: Supplementary file 1 [file nanomaterials-13-03072-s001.zip › nanomaterials-2733819-supplementary.pdf]

Supporting information

General Strategies for Preparing Hybrid Polymer/Quantum Dot Nanocomposites for Color Conversion

Guan-Hong Chen [1], Chen-Te Lin [2], Po-Hsun Chen [2], Tyng-Woei Jang [2] and Hsueh-Shih Chen [1,2,3,*]

[1] Department of Materials Science and Engineering, National Tsing Hua University, Hsinchu 30013, Taiwan; p01122334@gmail.com

[2] College of Engineering, National Tsing Hua University, Hsinchu 30013, Taiwan; chentelin@gapp.nthu.edu.tw (C.-T.L.); xenic0413@gmail.com (P.-H.C.); mse111031624@gapp.nthu.edu.tw (T.-W.J.)

[3] College of Semiconductor Research, National Tsing Hua University, Hsinchu 30013, Taiwan

* Correspondence: chenhs@mx.nthu.edu.tw

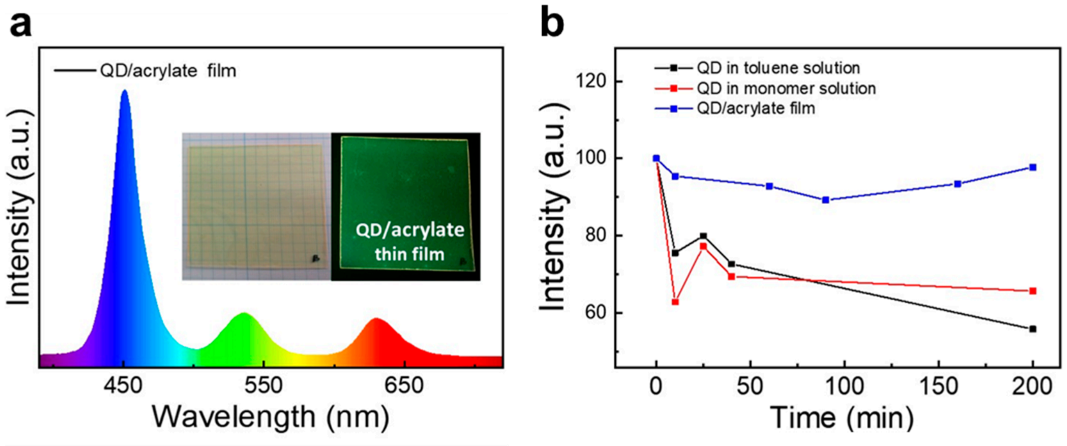

Figure S1. (a) PL spectrum of QD/acrylate optical film measured and integrated in a 15″ blue LCD backlit. Inset photos of QD/acrylate optical film under indoor light (left) and UV lamp (right). (b) Thermal stability of QD solution and QD/acrylate film.

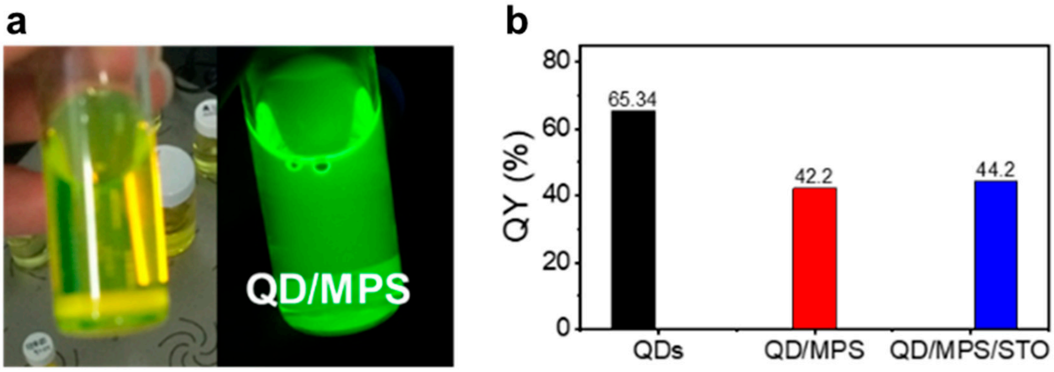

Figure S2. (a) Photos of MPS-modified QDs highly-dispersed in ethanol solution. (b) QYs of as-synthesized QDs, QD/MPS and QD/MPS/STO.

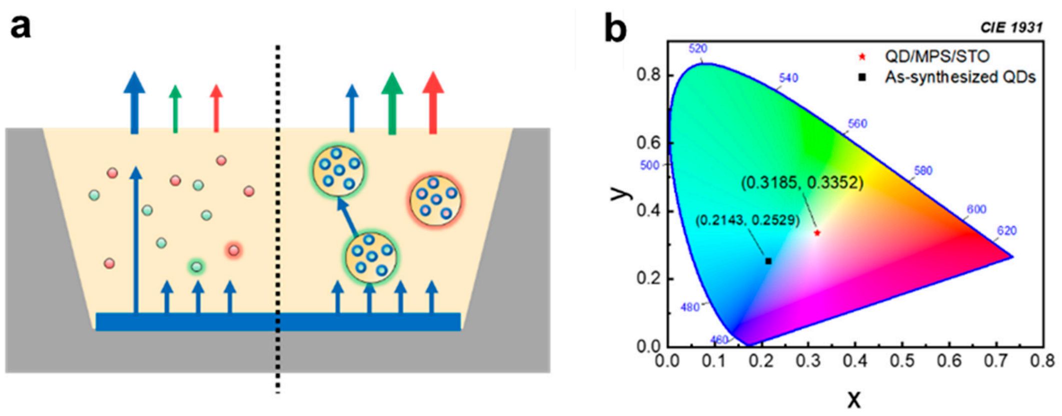

Figure S3. (a) Schematic diagram of QD optical diffusors for white LED compared with directly dispensing QDs in resin and showing longer light path length. (b) White points in CIE 1931 color space of QDCC-based on-chip LEDs along with the as-synthesis QDs (black point) and the QD optical diffusors (red point).

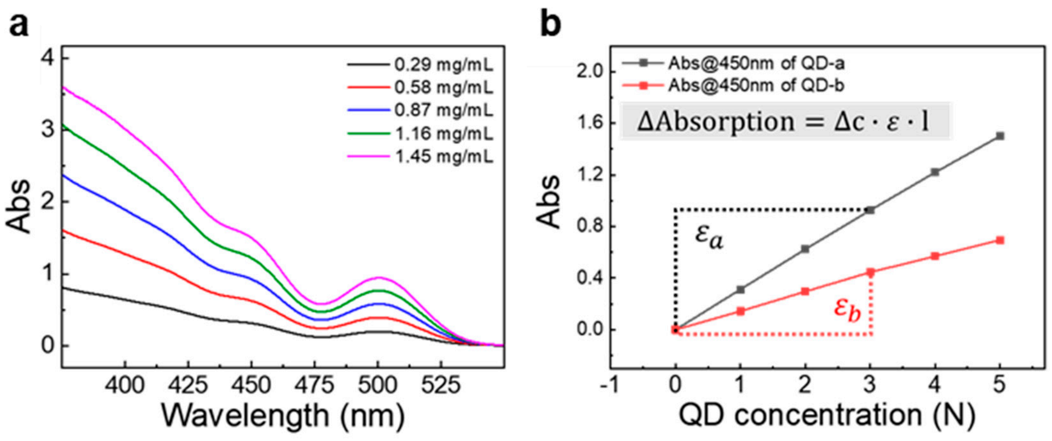

Figure S4. (a) Linear absorption evolution upon the increasing QD concentration of QD toluene solution. (b) Estimation of absorption coefficient for optical density of QDs.

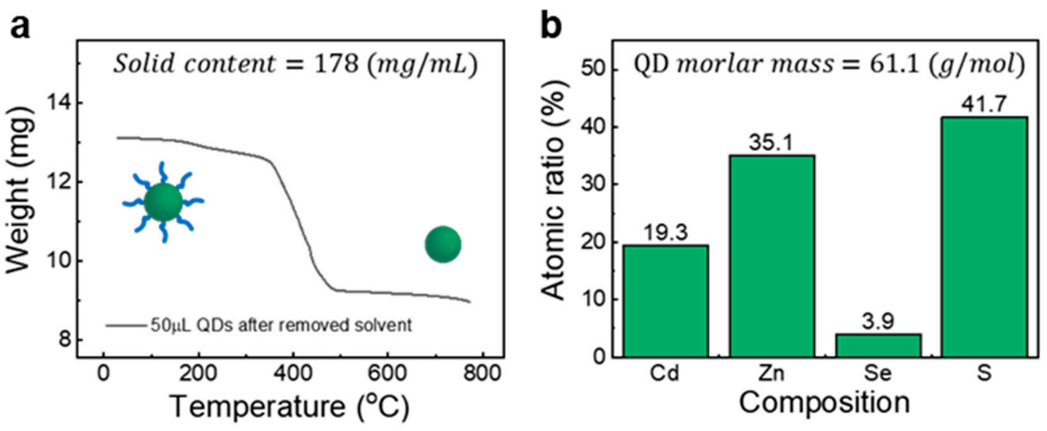

c

$$Molarity\ of\ QD\ solution\ (M) = \frac{178\ (g/mL)}{61.1\ (g/mol)} \cong 2900\ (mol/L)$$

Figure S5. Gravimetric quantification of QD solution using TGA and ICP-MS. (a) TGA measurement of the solid content of 50 uL QDs. The solvent is removed before TGA analysis. (b) Atomic ratio of QDs estimated from the ICP-MS. The QD molar mass is calculated according to the atomic ratio. (c) Calculation of QD molarity in solution by incorporating the data from TGA and ICP-MS.

Quantification of QD with gravimetric methods:

To quantify QD concentration by conventional gravimetric method, it requires the concentration of colloidal QD solution expressed by the solid content (denoted to QD weight) over solution volume (Figure S5a). However, the solid content of QDs comprised not only the inorganic crystalline structure but also the organic ligands shell having much boiling point (> 300 °C). The ligand content on QD is widely ranged from 20 wt. % to 50 wt. % depending on the different QDs, particle size, and elution method. This may add layers of difficulty on comparing the photonic property between the samples using different QDs, since the exact particle number is ambiguous. To measure the exact “solid content” of QD solution, an advanced gravimetric quantification is necessary. In most cases, the ligand is evaporated or decomposed by performing the thermogravimetry analysis (TGA) from room temperature to as high as 800 °C under inert atmosphere, which can separate the organic ligand or residue from the semiconductor core (Figure S5b). Hence, the amount of QDs dissolved in a specific volume of a liquid is acquired, of which the solution concentration of milligrams per milliliter (mg/mL) can be easily converted to part per million (ppm) for describing dilute solutions in chemistry (Figure S5c). In terms of scientific coherence, the unit of solid content from TGA can be further converted to the molar concentration (molarity, M), which is a unit of coherent system, by incorporating the atomic ratio estimated from an inductively coupled plasma mass spectrometry (ICP-MS).

Table S1. List of acrylic monomers used in this study and their solubility in water.

Abbreviation	Full name	Functional group(s)	Solubility in water (mg/L)
LMA	Lauryl methacrylate	Long-chain alkyl chain	Insoluble (< 0.001)
IBOA	Isobornyl acrylate	Bicyclic isobornyl group.	Insoluble (10)
LA	Lauryl acrylate	Long-chain alkyl chain.	Insoluble (0.15)
IOA	Isooctyl acrylate	Long-chain alkyl chain.	Insoluble (12)
DCPMA	Dicyclopentanyl Methacrylate	Dicyclopentanyl group.	Insoluble (N/A)
IDMA	Isodecyl methacrylate	Long-chain alkyl chain.	Insoluble (0.2)
MPEG600MA	Methoxy poly(ethylene glycol) methacrylate	Poly(ethylene oxide) segments.	Soluble (N/A)
HPPA	2-Hydroxy-3-phenoxypropyl acrylate	Hydroxyl and phenoxyl groups.	Soluble (3300)
$NP(PO)_2A$	Nonylphenol poly(propylene glycol) acrylate	Poly(propylene oxide) segments.	Soluble (N/A)
ACMO	4-Acryloylmorpholine	Acrylamide and morpholine groups.	Soluble (N/A)
GDMA	Glycerol dimethacrylate	Dimethacrylates.	Soluble (10250)
$NP(EO)_4A$	Nonylphenol poly(ethylene glycol)$_4$ acrylate	Poly(ethylene oxide) segments.	Soluble (N/A)
$NP(EO)_8A$	Nonylphenol poly(ethylene glycol)$_8$ acrylate	Poly(ethylene oxide) segments.	Soluble (N/A)
DEAA	N,N-diethylacrylamide	Acrylamide group.	Soluble (1000)
PHEA	2-Phenoxyethyl acrylate	Phenoxyethyl group.	Soluble (525)
BZA	Benzyl acrylate	Benzyl group.	Soluble (556)
THFA	Tetrahydrofurfuryl acrylate	Oxolane group.	Soluble (79000)

Abbreviation	Full name	Functional group(s)	Solubility in water (mg/L)
EOEOEA	2(2-Ethoxyethoxy) ethylacrylate	Di(ethylene oxide) segments.	Soluble (13430)
MPEG500MA	Methoxy poly(ethylene glycol) methacrylate	Poly(ethylene oxide) segments.	Soluble (N/A)
CTFA	Cyclic trimethylolpropane formal acrylate	Dioxane group.	Soluble (9300)

Note: The water solubility data of the monomers were required from product Information provide by chemical supplier.

Table S2. Changes in PL properties of green and red QDs dispersed in different acrylic monomers.

Acrylic monomer (short)	Solution QY (%)	Green PL peak (λ = 530 nm, FWHM = 30 nm)		Red PL peak (λ = 620 nm, FWHM = 26 nm)	
		Shifting	Broadening	Shifting	Broadening
LMA	61	+1	0	-1	0
IBOA	60	+1	0	0	+1
LA	60	+1	0	-1	0
Toluene	60	0	0	0	0
IOA	59	0	0	-2	+1
DCPMA	59	-1	0	+1	0
IDMA	59	0	0	+1	0
MPEG600MA	57	+2	0	+1	+1
HPPA	56	+3	0	+1	0
$NP(PO)_2A$	56	+4	-1	+1	+1
ACMO	56	-1	0	+1	+1
GDMA	54	+4	0	+2	+1
$NP(EO)_4A$	54	+3	-1	0	+1
$NP(EO)_8A$	53	+3	0	+1	+4
DEAA	51	+2	0	+1	0
PHEA	50	+2	-1	+1	+2
BZA	49	+5	-1	+1	+1
THFA	43	+1	-1	+1	0
EOEOEA	35	+5	-2	0	+1
MPEG500MA	23	+5	-2	-1	-1
CTFA	12	0	-1	+3	+3

Note: The wavelength and FWHM of the as-synthesized green and red QDs are measured when dispersed in toluene (gray marked row).
